# Integrative Approach Detected Association between Genetic Variants of microRNA Binding Sites of TLRs Pathway Genes and OSCC Susceptibility in Chinese Han Population

**DOI:** 10.1371/journal.pone.0101695

**Published:** 2014-07-07

**Authors:** Yun Wang, Chongkui Sun, Taiwen Li, Hao Xu, Yu Zhou, Hongxia Dan, Lu Jiang, Xin Zeng, Longjiang Li, Jing Li, Ga Liao, Qianming Chen

**Affiliations:** 1 State Key Laboratory of Oral Diseases, West China Hospital of Stomatology, Sichuan University, Chengdu, China; 2 West China School of Public Health, Sichuan University, Chengdu, China; Memorial University of Newfoundland, Canada

## Abstract

Oral squamous cell carcinoma (OSCC) is a leading malignancy worldwide; the overall 5-year survival rate is approximately 50%. A variety of proteins in Toll-like receptors (TLRs) pathway have been related with the risk of OSCC. However, the influence of genetic variations in TLRs pathway genes on OSCC susceptibility is unclear. Previous studies mainly focused on the coding region of genes, while the UTR region remains unstudied. In the current study, a bioinformatics approach was performed to select candidate single nucleotide polymorphisms (SNPs) on microRNA binding sites of TLRs pathway genes related with OSCC. After screening 90 OSCC related TLRs pathway genes, 16 SNPs were selected for genotyping. We found that rs5030486, the polymorphisms on 3′ UTR of *TRAF6*, was significantly associated with OSCC risk. AG genotype of *TRAF6* was strongly associated with a decreased risk of OSCC (OR = 0.252; 95% CI = 0.106, 0.598; p = 0.001). In addition, AG genotype was also related with a reduced risk of OSCC progression both in univariable analysis (HR = 0.303, 95% CI = 0.092, 0.995) and multivariable analysis (HR = 0.272, 95% CI = 0.082, 0.903). Furthermore, after detecting the mRNA expression level of *TRAF6* in 24 OSCC patients, we found that *TRAF6* expression level was significantly different between patients carrying different genotypes at locus rs5030486 (p = 0.013), indicating that rs5030486 of *TRAF6* might contribute to OSCC risk by altering *TRAF6* expression level. In general, these data indicated that SNP rs5030486 could be a potential bio-marker for OSCC risk and our results might provide new insights into the association of polymorphisms within the non-coding area of genes with cancers.

## Introduction

Oral squamous cell carcinoma (OSCC) is one of the top ten malignancies worldwide, with approximately 500, 000 new cases each year [1]. In addition to environmental factors including alcohol, tobacco, chronic inflammation and viral infection, genetic factors are also found to play an important role in the occurrence and progression of OSCC [2]. Although many genes and signal pathways have been associated with OSCC [3], the precise function of genetic variants in the development of OSCC remains unclear.

Chronic inflammation takes part in all the three mechanistic phases of carcinogenesis [4]–[6]. Several molecular pathways have been linked to the inflammatory response during malignant transformation, and the Toll-like receptors (TLRs) pathway is one of the most important of these pathways [7]. TLRs are a family of transmembrane proteins which play key roles in the innate immune system by recognizing pathogen associated molecular patterns (PAMPs), and then activating the adaptive immune system [8]. Previous studies have demonstrated the contribution of TLRs pathway to the general development of cancer [9]–[11], as well as increased risk in OSCC [12], [13].

MicroRNAs are a class of endogenous, non-coding small RNAs which contain 22∼25 nucleotides. They play important roles in post-transcriptional regulation of gene expression by binding complementary sites on the 3′ untranslated region (UTR) of target mRNAs. Their functional region is called the “seed region”, which contains 6∼8 nucleotides to degrade target mRNA or inhibit the translation [14], [15]. Binding affinity and specificity between microRNA and mRNA depend on base pairing. Therefore, single nucleotide polymorphisms (SNPs) in the 3′-UTR region of mRNA may modify the binding affinity of microRNA with mRNA [16]. Such a disruption would alter the gene expression and may influence cancer risk. Recent studies have confirmed the association between SNPs in microRNA binding site and cancer risk [17]–[19].

Previous studies in OSCC genetics mainly focused on the polymorphisms in the coding regions [20], [21]. However, the effect of non-coding SNPs in OSCC risk is unexplored. We hypothesize that polymorphisms in microRNA binding sites of genes in TLRs pathway genes might have influence on OSCC risk. In this study, SNPs on microRNA binding sites of Toll-like receptors (TLRs) pathway genes related to OSCC were selected using a bioinformatics approach. Afterwards, a hospital-based case-control study was then carried out to validate the association of candidate SNPs with OSCC susceptibility in Chinese Han Population.

## Materials and Methods

### 1 Study population

186 case subjects, clinically and pathologically diagnosed as OSCC from 2009 to 2012, was recruited for this study. 186 cancer-free subjects that were matched with case subjects by gender and age were recruited as controls. All subjects were identified from West China Hospital of Stomatology, Sichuan University (Chengdu, China). Clinical information was collected from all case subjects. A follow-up study was performed in all the enrolled OSCC patients until July 2013. 1ml peripheral blood sample was drawn from all subjects and stored at −80°C for further detection. Written informed consent was obtained from each subject.

### 2 Ethics statement

This study has been approved by the Ethnic Committee of State Key Laboratory of Oral Diseases, and the Clinical Trials Registration number is WCHSIEC-D-2012-00001.

### 3 SNPs selection

**Figure 1:**
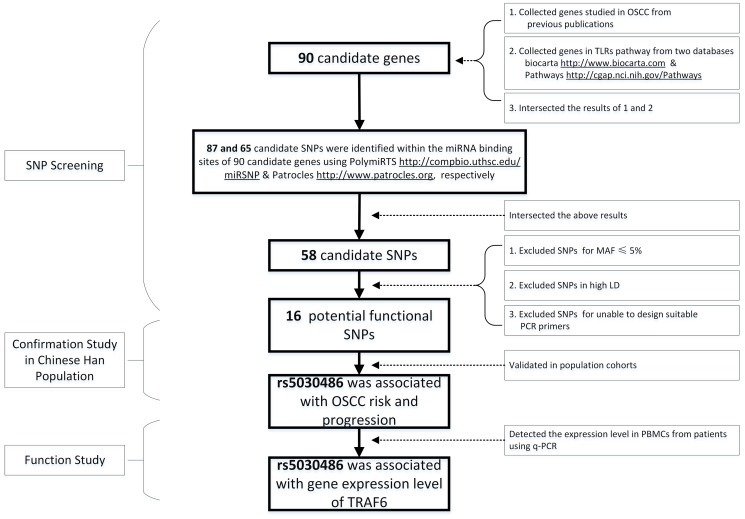
The overall schematic diagram of this study.

Extensive literature review and bioinformatical approaches were used to select SNPs within miRNA binding sites. Initially, we identified 90 OSCC-related genes involved in TLRs pathway by scanning related reviews from two databases in the past five years (http://www.biocarta.com, http://cgap.nci.nih.gov/Pathways). SNPs within the miRNA binding sites of these 90 genes were searched in two different databases. 87 and 65 SNPs within the miRNA binding sites of these 90 genes were identified in PolymiRTS (http://compbio.uthsc.edu/miRSNP; last updated in Apr. 2012) and Patrocles. (http://www.patrocles.org; last updated in Nov. 2010), respectively. The searching results were then intersected and 58 SNPs were selected. After checking the minor allele frequency (MAF) of these SNPs (http://www.ncbi.nlm.nih.gov/snp/), which was based on the frequencies in Asia population, 23 out of 58 were excluded with the frequency of no more than 5%. Then, LDSelect program (http://droog.gs.washington.edu/ldSelect.html) was used to separate the rest SNPs in order to exclude the SNP in high linkage disequilibrium and 7 SNPs were removed. In the remaining 28 SNPs, 12 were eliminated as their primers did not fit PCR or iPLEX condition. Finally, 16 SNPs were selected for genotyping. The flow diagram of selection was shown in Figure 1. Information for all selected genes and SNPs is shown in Table S1, Table S2 and Table S3.

### 4 DNA extraction and SNP genotyping

DNA was extracted from frozen peripheral blood samples using QIAmp DNA extraction kit (Qiagen) according to the manufacturer's protocol. Genotyping of chosen SNPs was done by Sequenom MassARRAY & iPLEX assay [22]. The primers of each chosen SNP for PCR and iPLEX assays are listed in Table 1.

**Table 1:** The information about six predicted polymorphisms.

Gene	SNP	Chromosome	Major/minor allele	PCR primers	iPLEX primers	Putative microRNAs
TRAF6	rs5030486	11	A/G	F 5′-ACGTTGGATGTACTGGCAAAATACTTTCC-3′ R 5′-ACGTTGGATGTCCAGCCATAACATTCTGTC-3′	CCAATTAAGTTCCAACCAGC	hsa-miR-138-5p
MYD88	rs6853	3	G/A	F 5′-ACGTTGGATGGCGTACAAAACATGTAGAAG-3′ R 5′-ACGTTGGATGCACCTGTCCCCCTTTAATAC-3′	CTCCGGGGCATTTTAAAGCCATCTC	hsa-miR-143
MAPK14	rs8510	6	C/T	F 5′-ACGTTGGATGGCATTTACAACCAAACATGGC-3′ R 5′-ACGTTGGATGCCTCATCTCCTTTATTGCAG-3′	CCCCGTTATTGCAGTTCAAATCCT	hsa-miR-541*
MAP3K7	rs2131906	6	A/G	F 5′-ACGTTGGATGTTTTCCTTTTCTCATGGTGG-3′ R 5′-ACGTTGGATGCTCATTCAAGTCACAGATGC-3′	ATGTTATGCAATGAAACAGTAAAA	hsa-miR-297, hsa-miR-548e, hsa-miR-548f
TLR6	rs5743823	4	T/C	5′-ACGTTGGATGGGAAATTCAACTTAAGAAACC-3′F R 5′-ACGTTGGATGCCTCCAGACAGTTACTTACG-3′	GAGGCAGTTACTTACGACTGTACT	hsa-miR-452
TLR4	rs7869402	9	C/T	F 5′-ACGTTGGATGACCTTCACACGTAGTTCTCC-3′ R 5′-ACGTTGGATGTTTAGGGAGACACAGATGGC-3′	TCCCTCCCCTGTACC	hsa-miR-539

### 5 Real-time quantitative PCR analysis

Total RNA was isolated from frozen peripheral blood of 24 OSCC patients using RiboPure-Blood Kit (Applied Biosystems) according to the manufacturer's instructions. First strand cDNA was generated by High Capacity cDNA Reverse Transcription kit (Applied Biosystems). Analysis of the cDNA was performed by Quantitative real-time PCR using SYBR Select Master Mix (Applied Biosystems) and ABI Prism 7900HT Sequence Detection System (Applied Biosystems). The primers for *TRAF6* were 5′ GGATTCTACACTGGCAAACCCG 3′ (forward) and 5′ CCAAGGGAGGTGGCTGTCATA 3′ (reverse). Endogenous *β-actin* was used as an internal control and the mRNA expression level of *TRAF6* was normalized by that of *β-actin* with the formula ratio of Ct *_TRAF6_*/Ct *_β-actin_* *100% [23].

To detect the miRNA expression level in blood samples, TaqMan MicroRNA Reverse Transcription Kit(Applied Biosystems) was used for transcription and TaqMan Universal Master Mix II(Applied Biosystems) was used for quantitative real-time PCR with the TaqMan MicroRNA primer sets (Applied Biosystems). The PCR was performed on the ABI Prism 7900HT Sequence Detection System (Applied Biosystems). U6, which is the most highly conserved and prevalent non-coding small nuclear RNA (snRNA) in vertebrate, was used as an internal control and the miRNA expression level of has-miR-138 was normalized by that of U6 with the formula ratio of Ct_miR-138_/Ct_U6_ *100% [23]. The analysis of both mRNA and miRNA expression level in peripheral blood of OSCC patients were performed by a quantification PCR experiment.

### 6 Statistical analysis

All statistical analysis in this study was performed using SPSS 19.0. For each selected polymorphism, Hardy–Weinberg equilibrium was determined to compare the observed frequencies of each genotype to the expected ones in control subjects by χ^2^ test. Student's t test was used to explore the difference in continuous variables such as distribution of age, mRNA and miRNA expression for subjects. Pearson χ^2^ test or Fisher's exact test were used to analyze the relationship between patient characteristics and SNP genotypes when appropriate. The OSCC risk was expressed as odds ratios (ORs) and 95% confidence intervals (CIs). The Kaplan–Meier analysis and Cox proportional hazards regression were utilized to estimate the possible association between the genotypes and progression-free survival time, which was calculated from the surgery day until the date of reoccurrence or death. All *p*-value was reported 2-sided and p-value ≤0.05 was considered to be statistically significant.

## Results

### 1 Characteristics of the study subjects

The clinicopathological characteristics of subjects recruited in this study were listed in Table 2. No significant difference was detected in the distribution of age (*p* = 0.175) and gender (*p* = 0.397) between patients and controls. According to the clinical information, the percentage of patients in clinical stageI&II(54.84%) were slightly higher than those in clinical stage III&IV (45.16%). Patients with higher histologic grade tumor were more than those with lower grade tumor (56.99% to 43.01%). The number of patients who did not have lymph node metastasis was two times more than those with metastasis (68.82% to 31.18%).

**Table 2:** Clinicopathological Characteristics of all the enrolled subjects

Characteristics	Subgroups	Case subjects (%) (N = 186)	Control subjects (%) (N = 186)	*p*-value
Age	Mean(±SD)	58.04(±12.810)	56.42(±9.882)	0.175
Gender	Male	116(62.37)	108(58.06)	
	Female	70(37.63)	78(41.94)	0.397
Clinical stage	I	41(22.04)		
	II	61(32.80)		
	III	42(22.58)		
	IV	42(22.58)		
Histological grade	1	106(56.99)		
	2	67(36.02)		
	3	13(6.99)		
Lymph node metastasis	Negative	128(68.82)		
	Positive	58(31.18)		

### 2 SNPs identification

Following the process described above, 16 SNPs from TLRs pathway genes were chosen for genotyping. Among them, 8 SNPs were identified with only one genotype in all cases, 2 SNPs showed statistical departure from Hardy–Weinberg equilibrium (*p*<0.05). These 10 SNPs were thus excluded from further analysis. Information of the remaining 6 SNPs was shown in Table 1.

### 3 Association between SNPs and OSCC susceptibility

The genotype and allele frequencies of 6 selected SNPs in all subjects were listed in Table 3. The genotype and allele frequencies of SNP rs5030486 were significantly different between case and control subject and might be associated with OSCC risk. Compared with the AA genotype, AG genotype was strongly associated with a decreased risk of OSCC (OR = 0.252; 95% CI = 0.106, 0.598; p = 0.001). The frequency of A allele is significantly higher than the G allele in the cancer subjects than the controls (OR = 0.266; 95% CI = 0.114, 0.623; p = 0.001). No significant association with the risk of OSCC was detected among the other 5 polymorphisms.

**Table 3:** Association between SNPs and the risk of OSCC.

Gene name	Polymorphism		Case (N = 186)		Control (N = 186)		OR (CI 95%)	*p*-value
			N	%	N	%		
TRAF6	rs5030486	AA	179	96.24	161	86.56	1 ref	
		AG	7	3.76	25	13.44	0.252(0.106,0.598)	0.001
		A	365	98.12	347	93.28	1 ref	
		G	7	1.88	25	6.72	0.266(0.114,0.623)	0.001
MYD88	rs6853	AA	170	91.40	174	93.55	1 ref	
		AG	16	8.60	12	6.45	1.365(0.627,2.970)	0.432
		A	356	95.70	360	96.77	1 ref	
		G	16	4.30	12	3.23	1.348(0.629,2.891)	0.441
MAPK14	rs8510	CC	52	27.96	41	22.04	1 ref	
		CT	77	41.40	99	53.23	0.613(0.370,1.017)	0.058
		TT	57	30.65	46	24.73)	0.977(0.556,1.718)	0.936
		CT+TT	134	72.05	145	77.96	0.729(0.455,1.168)	0.188
		C	181	48.77	181	48.77	1 ref	
		T	191	51.23	191	51.23	1.000(0.750,1.333)	1.000
MAP3K7	rs2131906	AA	174	93.55	178	95.70	1 ref	
		AG	12	6.45	8	4.30	1.534(0.612,3.845)	0.358
		A	360	96.77	364	97.85	1 ref	
		G	12	3.23	8	2.15	1.517(0.613,3.754)	0.365
TLR6	rs5743823	TT	184	98.92	185	99.46	1 ref	
		TC	2	1.08	1	0.54	2.011(0.181,22.369)	1.000
		T	370	99.46	371	99.73	1 ref	
		C	2	0.54	1	0.27	2.005(0.181,22.212)	1.000
TLR4	rs7869402	CC	156	83.87	157	84.41	1 ref	
		CT	27	14.52	29	15.59	0.937(0.530,1.655)	0.823
		TT	3	1.61	0	0.00	NA	0.248
		CT+TT	30	16.13	29	15.59	1.041(0.597,1.816)	0.887
		C	339	91.13	343	92.2	1 ref	
		T	33	8.87	29	7.80	1.151(0.684,1.938)	0.596

OR, odds ratio; CI, confidence interval.

The bold numbers mean the *p*-value is less than 0.05.

### 4 Association between SNPs and OSCC progression

**Figure 2:**
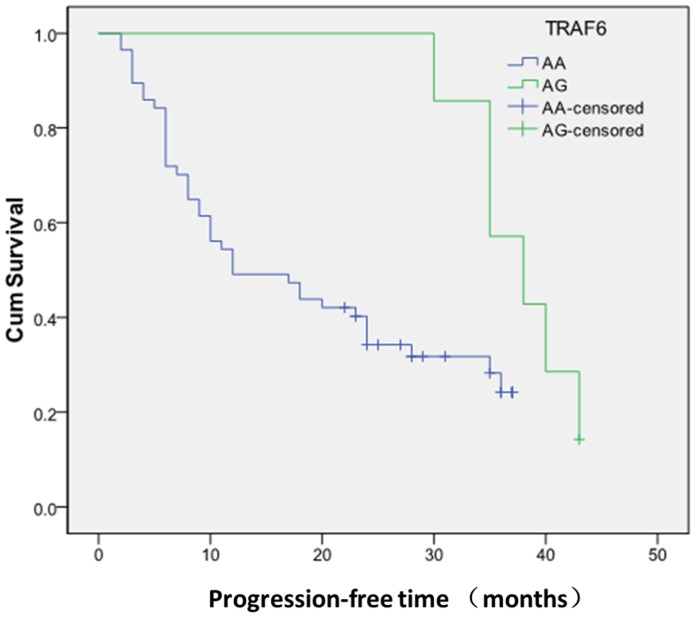
Kaplan–Meier curves of progression-free survival time of OSCC patients carrying AA and AG genotypes of rs5030486 on *TRAF6*.

Until July, 2013, 70 OSCC patients among all the enrolled case subjects completed the follow-up study and 46 of them suffered from OSCC progression. After comparing the clinicopathological features of the entire cohort and the survival analysis sub-cohort, no significant difference was found in the clinicopathological of these two cohorts. Thus, the sub-cohort could be the representative of the entire cohort (Table S4). The median time for follow-up and progression-free survival was 23±13.607 months and 10±12.065 months, respectively. The relationship between OSCC progression and the 6 selected polymorphisms were summarized in Table 4. Patients carrying AG genotype of rs5030486 had longer progression-free survival time, and their risk of cancer progression was 0.303 times comparing with those carrying wild type genotype AA (HR = 0.303, 95% CI = 0.092, 0.995) (Figure 2). Moreover, after a multivariable analysis adjust for age, gender, clinical stage, histologic grade and lymph node metastasis of OSCC patients, polymorphism rs5030486 was found to be related with progression-free survival independent of other variables (HR = 0.272, 95% CI = 0.082, 0.903). No significant association with OSCC progression was observed in the remaining 5 polymorphisms.

**Table 4:** Progression-free survival analysis of the selected six SNPs in OSCC patients

Polymorphisms	Genotypes	N (all, N = 64)		N (reoccurrence, N = 46)		*HR (95% CI)	**HR (95% CI)
		N	%	N	%		
TRAF6 rs5030486	AA	57	89.1	40	70.2	1 ref	1 ref
	AG	7	10.9	6	85.7	0.303(0.092,0.995)	0.272(0.082,0.903)
MYD88 rs6853	AA	58	90.6	44	75.9	1 ref	1 ref
	AG	6	9.4	2	33.3	0.265(0.063,1.109)	0.328(0.097,1.109)
MAPK14 rs8510	TT	16	25.0	11	68.8	1 ref	1 ref
	CT	32	50.0	23	71.9	1.060(0.514,2.186)	1.221(0.578,2.577)
	CC	16	25.0	12	75.0	0.759(0.324,1.777)	0.811(0.318,2.068)
MAP3K7 rs2131906	AA	63	98.4	46	73.0	1 ref	1 ref
	AG	1	1.6	0	0.0	0.048(0.000,1470.229)	0.794(0.175,3.599)
TLR6 rs5743823	TT	63	98.4	45	71.4	1 ref	1 ref
	CT	1	1.6	1	100.0	1.442(1.197,10.570)	1.520(0.194,11.932)
TLR4 rs7869402	TT	1	1.6	1	100.0	1 ref	1 ref
	CT	8	12.5	8	100.0	5.892(0.645,53.845)	11.792(0.949,146.530)
	CC	55	85.9	37	67.3	2.579(0.320,21.077)	2.802(0.251,31.225)

HR  =  hazard ratio; CI  = confidence interval.

The bold numbers mean the *p*-value is less than 0.05.

* A univariable analysis.

** A multivariable analysis adjusted for age, gender, clinical stage, histologic grade and lymph node metastasis.

### 5 Correlation analysis between SNP rs5030486 and expression level of *TRAF6* mRNA

**Figure 3:**
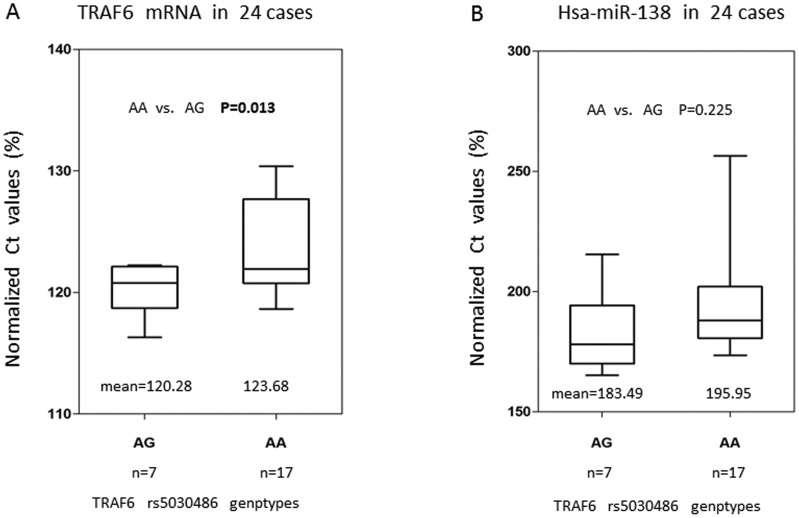
The expression level of *TRAF6* mRNA and hsa-miR-138 in PBMCs from 24 OSCC patients with known rs5030486 genotype. β-actin and U6 were used as the internal reference to normalize the Ct value of *TRAF6* mRNA and hsa-miR-138, respectively. Normalized Ct value represented the relative expression level. Lower normalized Ct value meant higher *TRAF6* or hsa-miR-138 expression. (A) Normalized Ct value of *TRAF6* mRNA was significant lower in AG genotype than that in AA genotype (p = 0.013), thus the expression level of *TRAF6* was higher in patients carrying rs5030486 AG genotype than those carrying AA genotype. (B) No statistical difference in normalized Ct value of hsa-miR-138 was detected between patients carrying rs5030486 AG genotype and AA genotype (p = 0.225).

The relationship of SNP rs5030486 and the mRNA expression level of *TRAF6* in peripheral blood mononuclear cell (PBMC) were analyzed in 24 OSCC patients. As shown in Figure 3, the normalized Ct values for patients carrying rs5030486 AG genotype (mean±SD: 120.28±2.36) were significant lower than those carrying AA genotype (mean±SD: 123.68±3.87) (p = 0.013), indicating cases with AG genotype had higher level of *TRAF6* expression than those with AA genotype. The expression level of the other 5 genes was not examined as their SNPs did not show any significant association with OSCC risk in this study.

Using both TargetScan (http://www.targetscan.org; version 6.2) and PolymiRTS (http://compbio.uthsc.edu/miRSNP; last updated in Apr. 2012), we found that hsa-miR-138 was a putative microRNA which could bind to rs5030486 of *TRAF6*. Our hypothesis was that the mRNA-miRNA binding affinity would be different when miR-138 bound to different rs5030486 genotypes, and the difference of affinity might change the expression of *TRAF6*. In order to validate this hypothesis, we tried to exclude the possibility that patients with different rs5030486 genotypes might have different miR-138 levels, which might contribute to the change of *TRAF6* expression. The expression level of miR-138 was examined in PBMCs from 24 OSCC patients by real-time quantitative PCR. As shown in Figure 3, no statistical difference in normalized Ct values of miR-138 was found between patients with rs5030486 AG (mean±SD: 183.49±17.37) and AA genotypes (mean±SD: 195.95±23.77) (p = 0.225).

## Discussion

A number of studies have reported the contribution of TLRs genes to the development and progression of OSCC [24], [25]. However, the effect of SNPs in microRNA binding sites of TRLs pathway genes on OSCC predisposition is still unknown. In this study, we selected 16 SNPs by methodically screening the miR-SNPs in OSCC related TLRs pathway genes, and then performed a hospital-based case-control study to explore the association between these SNPs and OSCC risk. We found that rs5030486,the polymorphisms on 3′ UTR of *TRAF6*, had significant association with OSCC risk.

TRAF6 (Tumor necrosis factor receptor-associated factors 6) is an adaptor protein capable of interacting with a variety of cell surface receptors. [26]. As an important adaptor protein, TRAF6 is involved in various signaling pathways and cell behaviors [27], [28]. Recent studies have shown that TRAF6 plays an important role in cancer pathogenicity [29], [30]. Based on previous studies about the function of TRAF6 in other cancers, we hypothesized that TRAF6 might also contribute to the initiation and development of OSCC. In this study, our data demonstrated a potential association between rs5030486 of *TRAF6* and OSCC risk in Chinese Han Population. To our knowledge, this study is the first to explore the relationship between a *TRAF6* polymorphism and OSCC risk.

Although *TRAF6* has been demonstrated to play a key role in a variety of cellular processes [31], [32], information about the expression and function of *TRAF6* in vivo is still unknown. Up to date, few studies have investigated the prognostic role of *TRAF6* in human cancers. Paik et al demonstrated that the miR-146a, which targeted *TRAF6* directly, could predict the prognosis of NK/T-cell lymphoma [33]. In another study, the *TRAF6* was correlated with the progression-free survival of NSCLC patients, concordant with the expression of *BRCA1/AEG-1* [34]. To the best of our knowledge, the potential prognostic value of *TRAF6* in OSCC has never been clarified before. In this study, we found that AG genotype of SNP rs5030486, located in 3′UTR of *TRAF6*, was correlated with a longer progression–free time of OSCC. In addition, the results of multivariate analysis showed that SNP rs5030486 could be used as an independent prognostic factor for progression–free survival. This is the first study exploring the association between *TRAF6* and progression of OSCC.

Disruption of mRNA-miRNA interactions could cause abnormal changes in mRNA expression, potentially leading to cancer development and progression. As rs5030486 was predicted to be located in a miRNA binding site, we expected this SNP would cause a change in *TRAF6* expression. After analyzing the mRNA level of T*RAF6* from PBMC in 24 OSCC patients, we found a significant difference in *TRAF6* expression between patients carrying AG and AA genotypes. Meanwhile, expression level of hsa-miR-138, which was predicted to bind to rs5030486, showed no difference between patients carrying AG and AA genotypes. Therefore, it is implied that the change of *TRAF6* expression was caused by the base transformation from A to G at SNP rs5030486 other than variation of miR-138 expression.

TRAF6 is an essential adaptor protein which is recruited by MyD88 in the TRL4/IL-1R pathway. So far, 11 TLR members have been identified, and TRL4 is the best-characterized member of this family. TLR4 can trigger the cascade reaction to activate two important transcription factors, NF-κB and AP-1, after recognizing the lipopolysaccharides (LPS) by recruiting adaptors such as MyD88, IRAK, TRAF6 and MAP3K7 [35]. NF-κB and AP-1 play key roles in cancer development by regulating the proliferation, differentiation, apoptosis and metastasis of cancer cells [36]. These two transcription factors were also found to contribute to the development and progression of OSCC [37], [38]. Given the critical function of TRAF*6* in activating the NF-κB and AP-1 pathways, it is not surprising that genetic variations in *TRAF6* gene could affect the risk and progression of OSCC. As this genetic variation resides in the miRNA-binding site, it may regulate the pathways by influencing the transcription and translation of the TRL4/IL-1R pathway gene.

In this study, we provided evidence for the potential association of SNP rs5030486 within the 3′ UTR of *TRAF6* with OSCC susceptibility and progression in Chinese Han population. Moreover, the existence of this SNP could lead to the change of *TRAF6* expression level in PBMCs of OSCC patients. Based on these results, we demonstrated for the first time that SNP rs5030486 could influence the risk of OSCC. This study also demonstrated the feasibility of the bioinformatics approach we used to identify candidate disease-related miR-SNPs. However, there are some limitations of this study. In order to elucidate the exact mechanism by which SNP rs5030486 affects *TRAF6* expression, further functional experiments are still required to detect the protein level of *TRAF6* in OSCC patient carrying different rs5030486 genotypes. In addition, further studies are needed to understand the effect that SNP rs5030486 has on the binding affinity between putative miRNA and *TRAF6*. Furthermore, replicating case-control studies on a larger scale and longer-time follow-up study are also required to verify the possible association between SNP rs5030486 of *TRAF6* and OSCC risk and progression. In conclusion, we have revealed new insights into the association of polymorphisms within the non-coding area of genes with OSCC. The finding in the study might also provide a potential biomarker for the diagnostic and prognostic of OSCC in future.

## Supporting Information

Table S1 **90 candidate genes in the SNPs selection flow.**
(DOCX)

Table S2 **58 candidate SNPs in the SNPs selection flow.**
(DOCX)

Table S3 **16 potential functional SNPs in the SNPs selection flow.**
(DOCX)

Table S4 **Demographic characteristics in the entire cohort and survival analysis sub-cohort.**
(DOCX)
