# Supplementary material for: Integrative Approach Detected Association between Genetic Variants of microRNA Binding Sites of TLRs Pathway Genes and OSCC Susceptibility in Chinese Han Population
Source: PLoS One. 2014 Jul 7;9(7):e101695. doi: 10.1371/journal.pone.0101695 (PMC4085003; doi:10.1371/journal.pone.0101695)
Supplement: Table S1 — 90 candidate genes in the SNPs selection flow. (DOCX) [file pone.0101695.s001.docx]

Supplementary Table 1. 90 candidate genes in the SNPs selection flow.

| **Gene ID** | **Gene Symbol** | **Gene Full Name** | **Location** | **SNPs identified by PolymiRTs** | **SNPs identified by Patrocles** |
| --- | --- | --- | --- | --- | --- |
| 801 | CALM1 | calmodulin 1 (phosphorylase kinase, delta) | 14q32.11 |  |  |
| 805 | CALM2 | calmodulin 2 (phosphorylase kinase, delta) | 2p21 |  |  |
| 808 | CALM3 | calmodulin 3 (phosphorylase kinase, delta) | 19q13.2-q13.3 | rs190593283, rs183554340 |  |
| 929 | CD14 | CD14 molecule | 5q31.1 | rs3776138 | rs3776138 |
| 1147 | CHUK | conserved helix-loop-helix ubiquitous kinase | 10q24-q25 |  |  |
| 1742 | DLG4 | discs, large homolog 4 (Drosophila) | 17p13.1 |  |  |
| 51295 | ECSIT | ECSIT signalling integrator | 19p13.2 |  |  |
| 1956 | EGFR | epidermal growth factor receptor | 7p12 |  |  |
| 5610 | EIF2AK2 | eukaryotic translation initiation factor 2-alpha kinase 2 | 2p22-p21 | rs181505633 |  |
| 2002 | ELK1 | ELK1, member of ETS oncogene family | Xp11.2 |  |  |
| 8772 | FADD | Fas (TNFRSF6)-associated via death domain | 11q13.3 |  | rs45542838 |
| 2353 | FOS | FBJ murine osteosarcoma viral oncogene homolog | 14q24.3 | rs186966778 |  |
| 2778 | GNAS | GNAS complex locus | 20q13.3 |  |  |
| 2782 | GNB1 | guanine nucleotide binding protein (G protein), beta polypeptide 1 | 1p36.33 |  |  |
| 2792 | GNGT1 | guanine nucleotide binding protein (G protein), gamma transducing activity polypeptide 1 | 7q21.3 |  |  |
| 2885 | GRB2 | growth factor receptor-bound protein 2 | 17q24-q25 |  |  |
| 2902 | GRIN1 | glutamate receptor, ionotropic, N-methyl D-aspartate 1 | 9q34.3 |  |  |
| 2903 | GRIN2A | glutamate receptor, ionotropic, N-methyl D-aspartate 2A | 16p13.2 |  |  |
| 2904 | GRIN2B | glutamate receptor, ionotropic, N-methyl D-aspartate 2B | 12p12 |  |  |
| 2905 | GRIN2C | glutamate receptor, ionotropic, N-methyl D-aspartate 2C | 17q25.1 |  |  |
| 2906 | GRIN2D | glutamate receptor, ionotropic, N-methyl D-aspartate 2D | 19q13.33 | rs112214473 |  |
| 3265 | HRAS | Harvey rat sarcoma viral oncogene homolog | 11p15.5 | rs45592334 | rs45592334 |
| 3480 | IGF1R | insulin-like growth factor 1 receptor | 15q26.3 |  |  |
| 3551 | IKBKB | inhibitor of kappa light polypeptide gene enhancer in B-cells, kinase beta | 8p11.2 | rs55829863, rs10108113 | rs10108113 |
| 3552 | IL1A | interleukin 1, alpha | 2q14 | rs140785926 |  |
| 3654 | IRAK1 | interleukin-1 receptor-associated kinase 1 | Xq28 | rs112750607, rs11556423, rs3027901 | rs11556423, rs3027901 |
| 3725 | JUN | jun proto-oncogene | 1p32-p31 | rs4647018, rs4647016, rs4647015 | rs12816, rs4647018 |
| 23643 | LY96 | lymphocyte antigen 96 | 8q21.11 |  |  |
| 5604 | MAP2K1 | mitogen-activated protein kinase kinase 1 | 15q22.1-q22.33 |  |  |
| 5605 | MAP2K2 | mitogen-activated protein kinase kinase 2 | 19p13.3 |  |  |
| 5606 | MAP2K3 | mitogen-activated protein kinase kinase 3 | 17q11.2 | rs2363192, rs2363187 | rs35380283, rs2363192, rs2363187 |
| 6416 | MAP2K4 | mitogen-activated protein kinase kinase 4 | 17p12 | rs189817203, rs35027510 | rs35027510 |
| 5608 | MAP2K6 | mitogen-activated protein kinase kinase 6 | 17q24.3 |  |  |
| 4214 | MAP3K1 | mitogen-activated protein kinase kinase kinase 1, E3 ubiquitin protein ligase | 5q11.2 |  |  |
| 9020 | MAP3K14 | mitogen-activated protein kinase kinase kinase 14 | 17q21 | rs1047841, rs180844357 | rs1047841 |
| 6885 | MAP3K7 | mitogen-activated protein kinase kinase kinase 7 | 6q15 | rs2131906, rs9451441, rs34631230, rs3734657, rs9396 | rs9396, rs2131906, rs9451441, rs3734657, rs34631230 |
| 5594 | MAPK1 | mitogen-activated protein kinase 1 | 22q11.21 | rs186857371, rs138946992, rs3810611, rs58437134, rs61757989 | rs3810611, rs58437134, rs61757989 |
| 1432 | MAPK14 | mitogen-activated protein kinase 14 | 6p21.3-p21.2 | rs8510, rs1803337 | rs1803337, rs8510 |
| 5595 | MAPK3 | mitogen-activated protein kinase 3 | 16p11.2 | rs3751867, rs11865228, rs113204102 | rs11865228, rs3751867, rs113204102 |
| 5599 | MAPK8 | mitogen-activated protein kinase 8 | 10q11.22 |  |  |
| 8569 | MKNK1 | MAP kinase interacting serine/threonine kinase 1 | 1p33 |  |  |
| 2872 | MKNK2 | MAP kinase interacting serine/threonine kinase 2 | 19p13.3 |  |  |
| 4609 | MYC | v-myc avian myelocytomatosis viral oncogene homolog | 8q24.21 |  |  |
| 4615 | MYD88 | myeloid differentiation primary response 88 | 3p22 | rs6853 | rs6853 |
| 4790 | NFKB1 | nuclear factor of kappa light polypeptide gene enhancer in B-cells 1 | 4q24 |  | rs35136542 |
| 4792 | NFKBIA | nuclear factor of kappa light polypeptide gene enhancer in B-cells inhibitor, alpha | 14q13 | rs45438895, rs187988391 |  |
| 4803 | NGF | nerve growth factor (beta polypeptide) | 1p13.1 |  |  |
| 4804 | NGFR | nerve growth factor receptor | 17q21-q22 |  |  |
| 4842 | NOS1 | nitric oxide synthase 1 (neuronal) | 12q24.22 |  |  |
| 5156 | PDGFRA | platelet-derived growth factor receptor, alpha polypeptide | 4q12 | rs147363356 |  |
| 8993 | PGLYRP1 | peptidoglycan recognition protein 1 | 19q13.2-q13.3 |  |  |
| 5465 | PPARA | peroxisome proliferator-activated receptor alpha | 22q13.31 |  |  |
| 5515 | PPP2CA | protein phosphatase 2, catalytic subunit, alpha isozyme | 5q31.1 |  |  |
| 5530 | PPP3CA | protein phosphatase 3, catalytic subunit, alpha isozyme | 4q24 |  |  |
| 5532 | PPP3CB | protein phosphatase 3, catalytic subunit, beta isozyme | 10q22.2 |  |  |
| 5533 | PPP3CC | protein phosphatase 3, catalytic subunit, gamma isozyme | 8p21.3 |  |  |
| 5567 | PRKACB | protein kinase, cAMP-dependent, catalytic, beta | 1p31.1 |  |  |
| 5568 | PRKACG | protein kinase, cAMP-dependent, catalytic, gamma | 9q13 |  |  |
| 5573 | PRKAR1A | protein kinase, cAMP-dependent, regulatory, type I, alpha | 17q24.2 |  |  |
| 5575 | PRKAR1B | protein kinase, cAMP-dependent, regulatory, type I, beta | 7p22 |  |  |
| 5576 | PRKAR2A | protein kinase, cAMP-dependent, regulatory, type II, alpha | 3p21.3-p21.2 |  |  |
| 5577 | PRKAR2B | protein kinase, cAMP-dependent, regulatory, type II, beta | 7q22 |  |  |
| 5578 | PRKCA | protein kinase C, alpha | 17q22-q23.2 |  |  |
| 5579 | PRKCB | protein kinase C, beta | 16p11.2 |  |  |
| 5801 | PTPRR | protein tyrosine phosphatase, receptor type, R | 12q15 | rs181431667, rs17108472, rs185619704 | rs17108472, rs185619704 |
| 5894 | RAF1 | v-raf-1 murine leukemia viral oncogene homolog 1 | 3p25 | rs5746251 | rs5746251 |
| 8737 | RIPK1 | receptor (TNFRSF)-interacting serine-threonine kinase 1 | 6p25.2 | rs17513574, rs185640889 | rs185640889 |
| 6195 | RPS6KA1 | ribosomal protein S6 kinase, 90kDa, polypeptide 1 | 1p | rs151125319, rs141078619 | rs151125319 |
| 9252 | RPS6KA5 | ribosomal protein S6 kinase, 90kDa, polypeptide 5 | 14q31-q32.1 | rs17127097, rs1286267, rs183140638 | rs17127097, rs1286267 |
| 6464 | SHC1 | SHC (Src homology 2 domain containing) transforming protein 1 | 1q21 |  |  |
| 6654 | SOS1 | son of sevenless homolog 1 (Drosophila) | 2p21 |  |  |
| 6714 | SRC | v-src avian sarcoma (Schmidt-Ruppin A-2) viral oncogene homolog | 20q12-q13 |  |  |
| 6774 | STAT3 | signal transducer and activator of transcription 3 (acute-phase response factor) | 17q21.31 |  |  |
| 10454 | TAB1 | TGF-beta activated kinase 1/MAP3K7 binding protein 1 | 22q13.1 |  |  |
| 23118 | TAB2 | TGF-beta activated kinase 1/MAP3K7 binding protein 2 | 6q25.1 | rs182985726, rs35859918, rs187462907, rs7896, rs34532338 | rs182985726, rs35859918, rs34532338 |
| 114609 | TIRAP | toll-interleukin 1 receptor (TIR) domain containing adaptor protein | 11q24.2 | rs625413, rs8177375 | rs625413 |
| 81793 | TLR10 | toll-like receptor 10 | 4p14 |  |  |
| 7097 | TLR2 | toll-like receptor 2 | 4q32 | rs190733702, rs35514550 | rs35514550, rs190733702 |
| 7098 | TLR3 | toll-like receptor 3 | 4q35 |  |  |
| 7099 | TLR4 | toll-like receptor 4 | 9q33.1 | rs35859918, rs1057313, rs7869402 | rs12533026, rs35859918, rs11536887, rs1064290, rs1057313, rs7869402 |
| 10333 | TLR6 | toll-like receptor 6 | 4p14 | rs5743823, rs5743829, rs55695972 | rs34400891, rs5743823, rs5743829 |
| 51284 | TLR7 | toll-like receptor 7 | Xp22.3 | rs10127190, rs12861036, rs5743784, rs5743786, rs80280330 | rs10127190, rs5743784, rs5743786, rs80280330 |
| 54106 | TLR9 | toll-like receptor 9 | 3p21.3 |  |  |
| 7124 | TNF | tumor necrosis factor | 6p21.3 | rs183157643, rs190947828, rs3093667, rs3093666, rs28501663 | rs3093667, rs190947828, rs3093666, rs28501663 |
| 7128 | TNFAIP3 | tumor necrosis factor, alpha-induced protein 3 | 6q23 | rs146085256, rs140042278 | rs146085256 |
| 7132 | TNFRSF1A | tumor necrosis factor receptor superfamily, member 1A | 12p13.2 |  |  |
| 7133 | TNFRSF1B | tumor necrosis factor receptor superfamily, member 1B | 1p36.22 |  |  |
| 54472 | TOLLIP | toll interacting protein | 11p15.5 | rs148701571, rs185077564, rs5744031, rs41314515 | rs148701571, rs5744031, rs41314515 |
| 8717 | TRADD | TNFRSF1A-associated via death domain | 16q22 | rs143536477, rs5744023 | rs5744023 |
| 7189 | TRAF6 | TNF receptor-associated factor 6, E3 ubiquitin protein ligase | 11p12 | rs5030486, rs11033658, rs56289909 | rs5030486, rs11033658, rs56289909 |
|  |  |  |  |  |  |
