# Supplementary material for: Integrative Approach Detected Association between Genetic Variants of microRNA Binding Sites of TLRs Pathway Genes and OSCC Susceptibility in Chinese Han Population
Source: PLoS One. 2014 Jul 7;9(7):e101695. doi: 10.1371/journal.pone.0101695 (PMC4085003; doi:10.1371/journal.pone.0101695)
Supplement: Table S2 — 58 candidate SNPs in the SNPs selection flow. (DOCX) [file pone.0101695.s002.docx]

Supplementary Table 2. 58 candidate SNPs in the SNPs selection flow.

| **Gene** | **SNP** | **Chromo-**  **some** | **Position** | **Major/Minor Allele** | **Putative Taget Site** | **Putative microRNAs** | |  |  |  |
| --- | --- | --- | --- | --- | --- | --- | --- | --- | --- | --- |
| CD14 | rs3776138 | 5 | 140011321 | C/G | TTAAAGAA | hsa-miR-302b, hsa-miR-302d | |  |  |  |
| HRAS | rs45592334* | 11 | 532310 | C/T | AGCTCCCC | hsa-miR-4300, hsa-miR-920, hsa-miR-5591-5p | |  |  |  |
| IKBKB | rs10108113* | 8 | 42308597 | T/C | TTTGTAAC | hsa-miR-615-5p | |  |  |  |
| IRAK1 | rs11556423** | X | 152929358 | G/T | TTTCAGGG | hsa-miR-587 | |  |  |  |
| IRAK1 | rs3027901 | X | 153277280 | A/G | ATCTGGAA | hsa-miR-488-5p | |  |  |  |
| JUN | rs4647018* | 1 | 59246581 | A/G | TTTACAGA | hsa-miR-3607-3p | |  |  |  |
| MAP2K3 | rs2363192** | 17 | 21158357 | G/A | CACCAGCA | hsa-miR-138 | |  |  |  |
| MAP2K3 | rs2363187* | 17 | 21158464 | G/A | AGTGCCTG | hsa-miR-181c | |  |  |  |
| MAP2K4 | rs35027510** | 17 | 11986899 | A/G | CAAGCACA | hsa-miR-636, hsa-miR-550, hsa-miR-218 | |  |  |  |
| MAP3K14 | rs1047841* | 17 | 43340631 | A/G | GCTGTCCA | hsa-miR-4520a-3p | |  |  |  |
| MAP3K7 | rs9396* | 6 | 91282232 | T/A | GATTTTTA | hsa-miR-548c-3p | |  |  |  |
| MAP3K7 | rs2131906 | 6 | 91282763 | A/G | ACATACAA | hsa-miR-297, hsa-miR-548e, hsa-miR-548f | |  |  |  |
| MAP3K7 | rs9451441*** | 6 | 91282764 | A/T | ACATACAA | hsa-miR-297 | |  |  |  |
| MAP3K7 | rs3734657*** | 6 | 91282929 | C/T | GACTGTTA | hsa-miR-194, hsa-miR-212, hsa-miR-132 | |  |  |  |
| MAP3K7 | rs34631230 | 6 | 91226286 | C/T | TACCAGCA | hsa-miR-138 | |  |  |  |
| MAPK1 | rs3810611** | 22 | 22115553 | A/G | ACCAGCAG | hsa-miR-138-5p | |  |  |  |
| MAPK1 | rs58437134*** | 22 | 22117065 | A/G | CCATGGAA | hsa-miR-5581-3p | |  |  |  |
| MAPK1 | rs61757989 | 22 | 22112868 | G/A | CCAGACAA | hsa-miR-632 | |  |  |  |
| MAPK14 | rs1803337** | 6 | 36185978 | C/T | ATTTCTCA | hsa-miR-539 | |  |  |  |
| MAPK14 | rs8510 | 6 | 36186158 | C/T | AATCCTTA | hsa-miR-541 | |  |  |  |
| MAPK3 | rs11865228 | 16 | 30033161 | G/T | TCATCTCA | hsa-miR-143 | |  |  |  |
| MAPK3 | rs3751867 | 16 | 30033353 | G/A | TCCCCACA | hsa-miR-491-5p | |  |  |  |
| MAPK3 | rs113204102** | 16 | 30128114 | G/C | CCACCCCA | hsa-miR-608 | |  |  |  |
| MYD88 | rs6853 | 3 | 38184370 | G/A | CATCTCAA | hsa-miR-143 | |  |  |  |
| PTPRR | rs17108472* | 12 | 71032068 | G/T | CTTCAGGA | hsa-miR-3140-5p | |  |  |  |
| PTPRR | rs185619704* | 12 | 71032198 | C/T | AAAGGGAA | hsa-miR-204-5p, hsa-miR-211-5p, hsa-miR-623 | |  |  |  |
| RAF1 | rs5746251* | 3 | 12625248 | C/T | TAGTCACA | hsa-miR-134, hsa-miR-3118 | |  |  |  |
| RIPK1 | rs185640889* | 6 | 3115246 | A/C | GGAGGGAG | hsa-miR-4469 | |  |  |  |
| RPS6KA1 | rs151125319* | 1 | 26900739 | A/C | GTCAGCAT | hsa-miR-4728-3p | |  |  |  |
| RPS6KA5 | rs17127097* | 15 | 91337463 | G/A | AAAGGGAA | hsa-miR-204-5p, hsa-miR-130b-5p | |  |  |  |
| RPS6KA5 | rs1286267* | 14 | 91337799 | A/G | ATTTGGGA | hsa-miR-186-3p | |  |  |  |
| TAB2 | rs182985726* | 6 | 149730902 | A/G | TCAAGAAA | hsa-miR-526b-5p, hsa-miR-578 | |  |  |  |
| TAB2 | rs35859918*** | 6 | 149731659 | C/A | ATGCCTTA | hsa-miR-124-3p, hsa-miR-3714, hsa-miR-506-3p | |  |  |  |
| TAB2 | rs34532338 | 6 | 149731141 | A/C | TATGTTAA | hsa-miR-302c | |  |  |  |
| TIRAP | rs625413* | 11 | 125669559 | C/T | AATGCCTT | hsa-miR-608 | |  |  |  |
| TLR2 | rs35514550** | 4 | 154846027 | T/G | GGTGCTGT | hsa-miR-559 | |  |  |  |
| TLR2 | rs190733702* | 4 | 154627110 | A/G | GTCCTGCC | hsa-miR-1286, hsa-miR-2682-5p, hsa-miR-34b-5p, hsa-miR-449c-5p | |  |  |  |
| TLR4 | rs35859918** | 9 | 149731659 | C/A | CAATGACT | hsa-miR-616-3p | |  |  |  |
| TLR4 | rs1057313* | 9 | 119517680 | G/T | ATTACCTC | hsa-miR-202 | |  |  |  |
| TLR4 | rs7869402 | 9 | 120478032 | C/T | CTTTCTCA | hsa-miR-539 | |  |  |  |
| TLR6 | rs5743823 | 4 | 38828649 | T/C | GAACAGTA | hsa-miR-452 | |  |  |  |
| TLR6 | rs5743829*** | 4 | 38827455 | C/T | CTGCCCCC | hsa-miR-4436b-3p | |  |  |  |
| TLR7 | rs10127190** | X | 12816916 | T/A | ATTTGCAC | hsa-miR-19a, hsa-miR-19b | |  |  |  |
| TLR7 | rs5743784* | X | 12907656 | T/C | TTCTCCTT | hsa-miR-4428 | |  |  |  |
| TLR7 | rs5743786** | X | 12817917 | T/C | CAGTTTTA | hsa-miR-548a-3p, hsa-miR-548e, hsa-miR-548f | |  |  |  |
| TLR7 | rs80280330 | X | 12907534 | A/C | ACACACAA | hsa-miR-147 | |  |  |  |
| TNF | rs3093667*** | 6 | 31653746 | G/T | TTTGCACT | hsa-miR-570 | |  |  |  |
| TNF | rs190947828* | 6 | 31545318 | A/G | GAGGAGGA | hsa-miR-660-3p | |  |  |  |
| TNF | rs3093666 | 6 | 31545733 | C/T | AGCCCTCC | hsa-miR-4721, hsa-miR-4446-3p | |  |  |  |
| TNF | rs28501663** | 6 | 31545828 | G/T | TTTGGGAG | hsa-miR-150-5p | |  |  |  |
| TNFAIP3 | rs146085256* | 6 | 138202536 | C/G | CCCCTCAG | hsa-miR-3184-5p | |  |  |  |
| TOLLIP | rs148701571* | 11 | 1296387 | A/G | GGCCTGAG | hsa-miR-1972, hsa-miR-4650-5p | |  |  |  |
| TOLLIP | rs5744031* | 11 | 1296618 | G/A | TCCATGAT | hsa-miR-490-5p | |  |  |  |
| TOLLIP | rs41314515 | 11 | 1298137 | C/A | GCCACCCC | hsa-miR-608 | |  |  |  |
| TRADD | rs5744023* | 11 | 1253928 | G/A | TGCCCAGA | hsa-miR-612, hsa-miR-1285 | |  |  |  |
| TRAF6 | rs5030486 | 11 | 36509146 | A/G | ACCAGCAG | hsa-miR-138-5p | |  |  |  |
| TRAF6 | rs11033658** | 11 | 36509510 | C/T | TTGCACTT | hsa-miR-130b | |  |  |  |
| TRAF6 | rs56289909*** | 11 | 36511067 | C/T | CAGAAGGT | hsa-miR-1237 | |  |  |  |
| * MAF was no more than 5% | |  |  |  |  |  |  | | |  |
| ** Primers did not fit PCR or iPLEX condition | | |  |  |  |  |  | |  | |
| *** SNPs in high linkage disequilibrium | | |  |  |  |  |  | |  | |
