# Supplementary material for: Integrative Approach Detected Association between Genetic Variants of microRNA Binding Sites of TLRs Pathway Genes and OSCC Susceptibility in Chinese Han Population
Source: PLoS One. 2014 Jul 7;9(7):e101695. doi: 10.1371/journal.pone.0101695 (PMC4085003; doi:10.1371/journal.pone.0101695)
Supplement: Table S3 — 16 potential functional SNPs in the SNPs selection flow. (DOCX) [file pone.0101695.s003.docx]

Supplementary Table 3. 16 potential functional SNPs in the SNPs selection flow.

| **Gene** | **SNP** | **Major/Minor Allele** | **Putative Taget Site** | **Putative microRNAs** | | **Detected Genotypes** | ***p-*Value for HWE Test** |
| --- | --- | --- | --- | --- | --- | --- | --- |
| CD14 | rs3776138* | C/G | TTAAAGAA | hsa-miR-302b, hsa-miR-302d | | CC | NA |
| IRAK1 | rs3027901* | A/G | ATCTGGAA | hsa-miR-488-5p | | GG | NA |
| MAP3K7 | rs2131906 | A/G | ACATACAA | hsa-miR-297, hsa-miR-548e, hsa-miR-548f | | AA, AG | 0.748 |
| MAP3K7 | rs34631230* | C/T | TACCAGCA | hsa-miR-138 | | CC | NA |
| MAPK1 | rs61757989* | G/A | CCAGACAA | hsa-miR-632 | | GG | NA |
| MAPK14 | rs8510 | C/T | AATCCTTA | hsa-miR-541 | | CC, CT, TT | 0.595 |
| MAPK3 | rs11865228* | G/T | TCATCTCA | hsa-miR-143 | | GG | NA |
| MAPK3 | rs3751867** | G/A | TCCCCACA | hsa-miR-491-5p | | GG, GA | 0.000 |
| MYD88 | rs6853 | G/A | CATCTCAA | hsa-miR-143 | | AA, AG | 0.441 |
| TAB2 | rs34532338* | A/C | TATGTTAA | hsa-miR-302c | | AA | NA |
| TLR4 | rs7869402 | C/T | CTTTCTCA | hsa-miR-539 | | CC, CT, TT | 0.210 |
| TLR6 | rs5743823 | T/C | GAACAGTA | hsa-miR-452 | | TT, TC | 0.972 |
| TLR7 | rs80280330** | A/C | ACACACAA | hsa-miR-147 | | AA, AC, CC | 0.000 |
| TNF | rs3093666* | C/T | AGCCCTCC | hsa-miR-4721, hsa-miR-4446-3p | | CC | NA |
| TOLLIP | rs41314515* | C/A | GCCACCCC | hsa-miR-608 | | CC | NA |
| TRAF6 | rs5030486 | A/G | ACCAGCAG | hsa-miR-138-5p | | AA,AG | 0.373 |
| * SNPs were identified with only one genotype in all cases | | | | |  |  |  |
| ** SNPs showed statistical departure from Hardy–Weinberg Equilibrium | | | | |  |  |  |
