# Supplementary material for: Integrative Approach Detected Association between Genetic Variants of microRNA Binding Sites of TLRs Pathway Genes and OSCC Susceptibility in Chinese Han Population
Source: PLoS One. 2014 Jul 7;9(7):e101695. doi: 10.1371/journal.pone.0101695 (PMC4085003; doi:10.1371/journal.pone.0101695)
Supplement: Table S4 — Demographic characteristics in the entire cohort and survival analysis sub-cohort. (DOCX) [file pone.0101695.s004.docx]

Supplementary Table 4. Demographic characteristics in the entire cohort and survival analysis sub-cohort.

| **Characteristics** | **Subgroups** | **Entire cohort (%)**  **(N=186)** | **Survival analysis sub-cohort(%)**  **(N=64)** | ***p*-value** |
| --- | --- | --- | --- | --- |
| Age | Mean(±SD) | 58.04(±12.810) | 56.97(±11.202) | 0.414 |
| Gender | Male | 116(62.37) | 40(62.50) | 0.985 |
|  | Female | 70(37.63) | 24(37.50) |  |
| Clinical stage | Ⅰ | 41(22.04) | 13(20.31) | 0.919 |
|  | Ⅱ | 61(32.80) | 21(32.81) |  |
|  | Ⅲ | 42(22.58) | 13(20.31) |  |
|  | Ⅳ | 42(22.58) | 17(26.56) |  |
| Histological grade | 1 | 106(56.99) | 40(62.50) | 0.677 |
|  | 2 | 67(36.02) | 21(32.81) |  |
|  | 3 | 13(6.99) | 3(4.69) |  |
| Lymph node metastasis | Negative | 128(68.82) | 44(68.75) | 0.992 |
|  | Positive | 58(31.18) | 20(31.25) |  |
